# Origin of Large Effective Phonon Magnetic Moments in Monolayer MoS_2_

**DOI:** 10.1021/acsnano.4c18906

**Published:** 2025-03-13

**Authors:** Hussam Mustafa, Cynthia Nnokwe, Gaihua Ye, Mengqi Fang, Swati Chaudhary, Jia-An Yan, Kai Wu, Connor J. Cunningham, Colin M. Hemesath, Andrew James Stollenwerk, Paul M. Shand, Eui-Hyeok Yang, Gregory A. Fiete, Rui He, Wencan Jin

**Affiliations:** † Department of Physics, Auburn University, Auburn, Alabama 36849, United States; ‡ Department of Electrical and Computer Engineering, Texas Tech University, Lubbock, Texas 79409, United States; § Department of Mechanical Engineering, Stevens Institute of Technology, Hoboken, New Jersey 07030, United States; ∥ Institute for Solid State Physics, The University of Tokyo, Chiba 277-8581, Japan; ⊥ Department of Physics, Astronomy, and Geosciences, Towson University, Towson, Maryland 21252, United States; # Department of Physics, University of Northern Iowa, Cedar Falls, Iowa 50614, United States; ∇ Department of Physics, Northeastern University, Boston, Massachusetts 02115, United States; ○ Quantum Sensing and Materials Institute, Northeastern University, Burlington, Massachusetts 01803, United States; ◆ Department of Physics, Massachusetts Institute of Technology, Cambridge, Massachusetts 02139, United States

**Keywords:** circularly polarized phonon, MoS_2_, phonon magnetic moment, helicity-resolved magneto-Raman spectroscopy, orbital-phonon coupling, spin fluctuation, 2D transition metal dichalcogenide

## Abstract

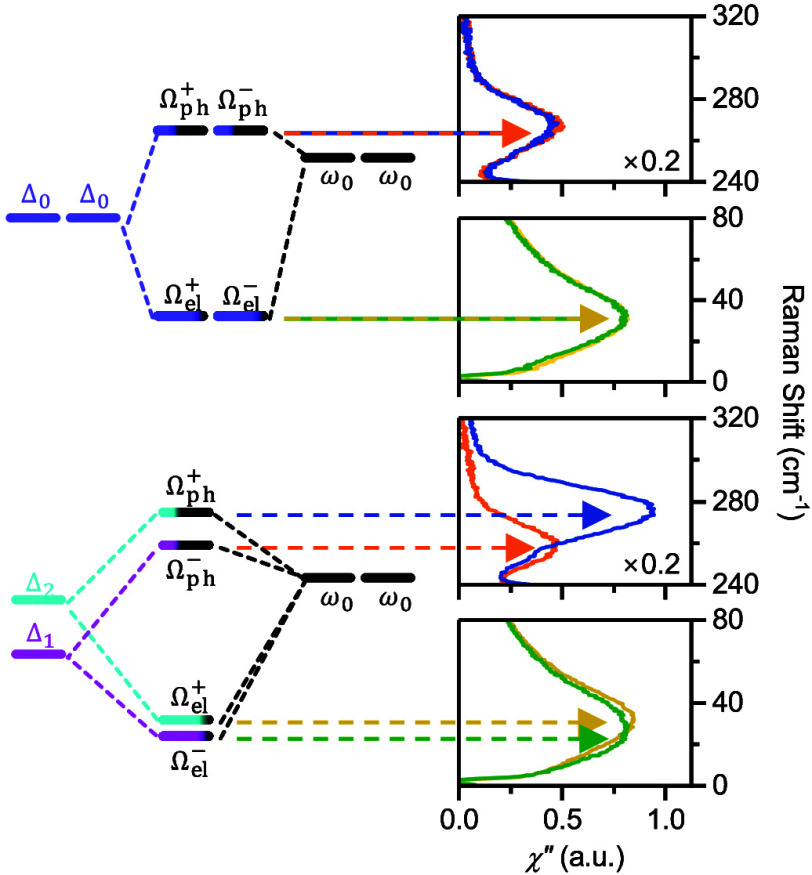

Recent helicity-resolved magneto-Raman spectroscopy measurement demonstrates large effective phonon magnetic moments of ∼2.5 μ_B_ in monolayer MoS_2_, highlighting resonant excitation of bright excitons as a feasible route to activate Γ-point circularly polarized phonons in transition metal dichalcogenides. However, a microscopic picture of this intriguing phenomenon remains lacking. In this work, we show that an orbital transition between the split conduction bands (Δ_0_ = 4 meV) of MoS_2_ couples to the doubly degenerate *E*″ phonon mode (Ω_0_ = 33 meV), forming two hybridized states. Our phononic and electronic Raman scattering measurements capture these two states: (i) one with predominantly phonon contribution in the helicity-switched channels and (ii) one with primarily orbital contribution in the helicity-conserved channels. An orbital-phonon coupling model successfully reproduces the large effective magnetic moments of the circularly polarized phonons and explains their thermodynamic properties. Strikingly, the Raman mode from the orbital transition is superimposed on a strong quasi-elastic scattering background, indicating the presence of spin fluctuations. As a result, the electrons excited to the conduction bands through the exciton exhibit paramagnetic behavior although MoS_2_ is generally considered as a nonmagnetic material. By depositing nanometer-thickness nickel thin films on monolayer MoS_2_, we tune the electronic structure so that the *A* exciton perfectly overlaps with the 633 nm laser. The optimization of resonance excitation leads to pronounced tunability of the orbital-phonon hybridized states. Our results generalize the orbital-phonon coupling model of effective phonon magnetic moments to material systems beyond the paramagnets and magnets.

Circularly polarized lattice vibration carries finite angular momentum, producing phonon magnetic moments.^1,2^ The frequencies of left- and right-handed circularly polarized phonons split in an external magnetic field, which is known as the phonon Zeeman effect. Beyond the picture of nuclear magneton phonon magnetic moments produced by the ionic charge current,^3,4^ sizable phonon Zeeman splitting has been observed in various material systems, including rare-earth halides,^5−8^ Dirac semimetals,^9^ narrow-gap semiconductors,^10^ topological crystalline insulators,^11^ perovskite materials,^12^ and magnets,^13−15^ which offers tremendous opportunities for manipulating the emergent magnetic phenomena of these materials.

To account for phonon magnetic moments up to the Bohr magneton (μ_B_) scale, one must consider additional contributions from electron–phonon coupling, which manifests itself in different forms. Theories based on nontrivial electronic band topology consider adiabatic evolution of the electronic states along the circularly polarized phonon modes that can induce electronic orbital magnetization.^16−21^ In parallel, theories based on spin-phonon coupling establish the coupling between phonon angular momentum and electron spin through the modification of crystal electric field^22^ or spin channels of doping-induced conduction electrons.^23−25^ In addition, an orbital-phonon coupling model^26^ was developed in paramagnetic and magnetic materials based on the hybridization of degenerate chiral phonon mode and degenerate orbital excitation. More generally, a recent proposal suggests that the role of electron–phonon coupling is to generate a non-Maxwellian field that leads to phonon-induced magnetic activity.^27^

Aside from these developments, studies of phonon magnetic moments in two-dimensional van der Waals materials remain scarce. Recently, helicity-resolved magneto-Raman spectroscopy measurements were carried out to study phonon magnetic moments in monolayer MoS_2_.^28^ When the laser wavelength [*hν* = 633 nm (1.96 eV)] is on resonance with the *A* exciton, a broad doubly degenerate Raman mode is identified at ∼267 cm^–1^ (33 meV). This mode obeys the helicity selection rule, exhibits giant phonon Zeeman splitting (∼2.5 μ_B_) and magnetic circular dichroism (∼50%) at ±7 T magnetic field, and is therefore assigned as a chiral phonon (or circularly polarized phonon).

Here, we propose the microscopic origin of the large phonon magnetic moment in MoS_2_. We show that the resonant excitation of the *A* exciton in monolayer MoS_2_ populates the electrons in the spin–orbit split conduction bands and enables an orbital transition between them. The coupling between the orbital transition and the *E*″ phonon mode produces two hybridized states. In addition to the previously reported 267 cm^–1^ (33 meV) mode in the helicity-switched channels, which corresponds to the phonon-dominated hybridized state, we find its orbital-dominated counterpart at 33 cm^–1^ (4 meV) in the helicity-conserved channels. Under an external magnetic field, both hybridized states split, and the giant effective phonon magnetic moment is attributed to the contribution from the sizable electron–phonon coupling between the orbital transition and the *E*″ phonon mode. The effective phonon magnetic moment gradually decays with elevated temperature as described by the orbital-phonon coupling model and vanishes at 200 K when the resonant condition breaks down. More importantly, the orbital transition is subject to strong spin fluctuations, as evidenced by the quasi-elastic scattering (QES) features in the low-frequency region of the electronic Raman response. The QES spectral intensity diverges at ∼40 K where the system has the maximum spin fluctuations. Therefore, electrons excited to the split conduction bands by the *A* exciton resemble paramagnetic behavior which can be described by Curie–Weiss law. Our results demonstrate that the orbital-phonon coupling model developed for paramagnets and magnets can be generalized to monolayer MoS_2_, establishing the exciton transition in spin-valley coupled transition metal dichalcogenides as a route for activating giant effective phonon magnetic moments in nonmagnetic materials.

## Results and Discussion

**Figure 1:**
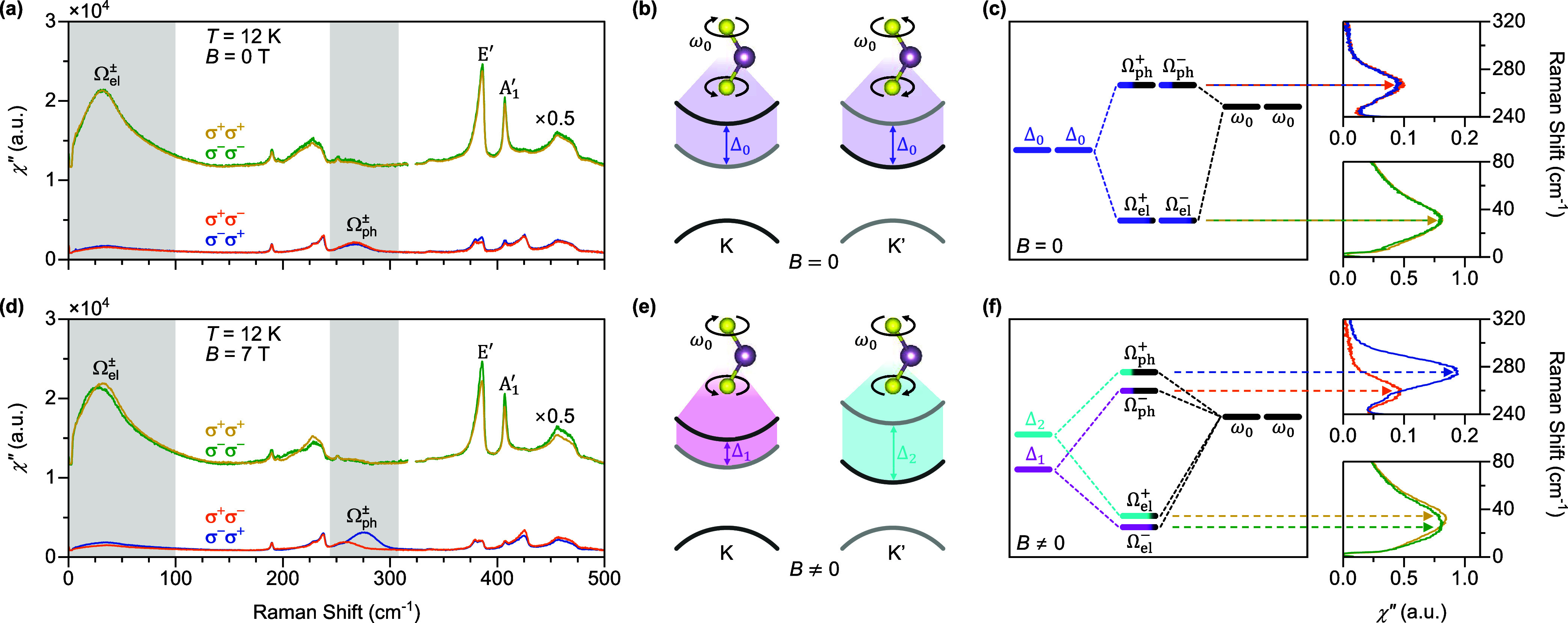
Orbital-phonon coupling model of MoS_2_. (a) Raman response χ″ of monolayer MoS_2_ in the helicity-conserved (σ^+^σ^+^/σ^–^σ^–^) and helicity-switched (σ^+^σ^–^/σ^–^σ^+^) channels. Ω_ph_^±^ and Ω_el_^±^ denote the hybridized states from orbital-phonon coupling with primarily phonon and electron contributions, respectively. (b) Schematic of *K* and *K*′ valleys with spin–orbit split conduction bands. Δ_0_ denotes the orbital transition between the split conduction bands and ω_0_ denotes the doubly degenerate *E*″ phonon mode. (c) Energy level diagram of the coupling between the orbital transition and the *E*″ phonon mode, producing hybridized states: the phonon-dominant state observed in the σ^+^σ^–^/σ^–^σ^+^ channels and the orbital-dominant state observed in the σ^+^σ^+^/σ^–^σ^–^ channels. (d) Raman response χ″ in helicity-resolved channels in 7 T magnetic field. (e) Schematic of low-energy (Δ_1_) and high-energy (Δ_2_) orbital transitions in external magnetic field. (f) Orbital-phonon coupling of MoS_2_ in an external magnetic field. The nondegenerate Δ_1_ and Δ_2_ orbital transitions produce the split Ω_el_^±^ and Ω_ph_^±^ modes, respectively. In (a) and (d), the Raman spectra in the σ^+^σ^+^/σ^–^σ^–^ channels in the 320–500 cm^–1^ range are scaled by a factor of 0.5.

In addition to lattice vibrations, Raman spectroscopy is a powerful tool to probe orbital states and the fluctuations associated with the charge/spin degrees of freedom through emergent features in the low-frequency region of the imaginary part of the Raman susceptibility, or Raman response χ″(ω), which is obtained from the measured Raman intensity divided by the Bose–Einstein factor [1 + *n*(ω)].^29,30^ We start with wide frequency range data of χ″(ω) in monolayer MoS_2_. Figure 1a,d shows χ″(ω) in helicity-conserved channels (σ^+^σ^+^ and σ^–^σ^–^) and helicity-switched channels (σ^+^σ^–^ and σ^–^σ^+^) at 0 and 7 T magnetic fields, respectively. At 0 T, a doubly degenerate mode labeled as Ω_ph_^±^ is observed at around 267 cm^–1^ (33 meV) in the helicity-switched channels, which is the circularly polarized phonon mode reported previously.^28^ In the low-frequency region, another doubly degenerate mode labeled as Ω_el_^±^ is observed at around 33 cm^–1^ (4 meV) in the helicity-conserved channels. This low-frequency broad mode was previously seen in exfoliated MoS_2_ flakes in linear polarization channels.^31^ The energy of the 33 cm^–1^ (4 meV) mode matches the splitting energy of the conduction bands of MoS_2_ due to spin–orbit coupling.^32^ Thus, we consider the origin of the Ω_el_^±^ mode to be the orbital transition between the split conduction bands (denoted as Δ_0_ in Figure 1b). Since the split conduction bands are a Kramers pair derived from the Mo *d*_*z*^2^_ orbitals,^33^ the orbital transitions have two copies associated with the *K* and *K*′ valleys. Also, the orbital transitions carry zero orbital angular momentum (Δ*m*_*l*_ = 0), which is consistent with their presence in helicity-conserved channels. As shown in Figure 1c, the doubly degenerate orbital transition (Δ_0_) couples to the doubly degenerate phonon mode (ω_0_), producing hybridized orbital-phonon modes with mainly orbital (Ω_el_^±^) and phononic (Ω_ph_^±^) contribution, respectively. When the Kramers degeneracy is lifted by applying an external magnetic field,^34^ the orbital transitions split into Δ_1_ = Δ_0_ – γB and Δ_2_ = Δ_0_ + γB (see Figure 1e). The valley Zeeman shift between *K* and *K*′ valleys^35,36^ is not shown in the band diagram as they are irrelevant to the orbital-phonon coupling. As shown in Figure 1f, at 7 T, the split Δ_1_ and Δ_2_ orbital transitions are reflected in the split Ω_el_^±^ modes, in which the 16 cm^–1^ splitting energy between Ω_ph_^+^ and Ω_ph_^–^ corresponds to an effective phonon magnetic moment of 2.45 μ_B_. In addition, the distinct intensity of the Ω_ph_^+^ and Ω_ph_^–^ was previously simulated by first-principles calculation of the Raman susceptibility.^28^ In the orbital-phonon coupling model, the imbalanced populations of the *A* exciton in the *K* and *K*′ valley under an external magnetic field (the higher-energy valley has larger *A* exciton population^35^) also contribute to the stronger intensity of the high-frequency Ω_ph_^+^ mode.

To quantify the orbital-phonon coupling model, we evaluate the magnitude of the effective phonon magnetic moment by calculating the saturated Zeeman splitting, which is defined as the energy difference between the left- and right-handed phonons normalized by the degenerate phonon energy^26,37^
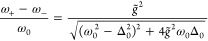
1where *g̃* is the coupling strength between the phonon mode and the orbital transition. Our first-principles calculation shows that only the *E*″ mode exhibits sizable coupling with the split conduction bands (*g̃* = 8 meV), while other phonon modes (*E*′, *A*_1_^′^, and *A*_2_^″^) possess negligible coupling (*g̃* = 10^–6^ meV). Details of the calculation are described in Supporting Information Section 1. Based on *g̃* = 8 meV, ω_0_ = 33 meV, and Δ_0_ = 4 meV,^32,38,39^ our model yields (ω_+_ – ω_–_)/ω_0_ = 5.88% for the Ω_ph_^±^ mode, which corresponds to 2.4 μ_B_ effective phonon magnetic moment and perfectly agrees with our measured value (2.45 μ_B_) at 12 K. In contrast, *E*′, *A*_1_^′^, and *A*_2_^″^ modes show the absence of phonon magnetic moments.^23^ In monolayer MoS_2_, the *E*″ phonon frequency is much larger than the orbital transition energy (ω_0_ ≫ Δ_0_); thus, the saturated Zeeman splitting can be estimated using (ω_+_ – ω_–_)/ω_0_ ≈ (*g̃*/ω_0_)^2^, that is, a second order effect.^37^ In this scenario, the sizable electron–phonon coupling strength plays a key role in producing large effective phonon magnetic moments.

**Figure 2:**
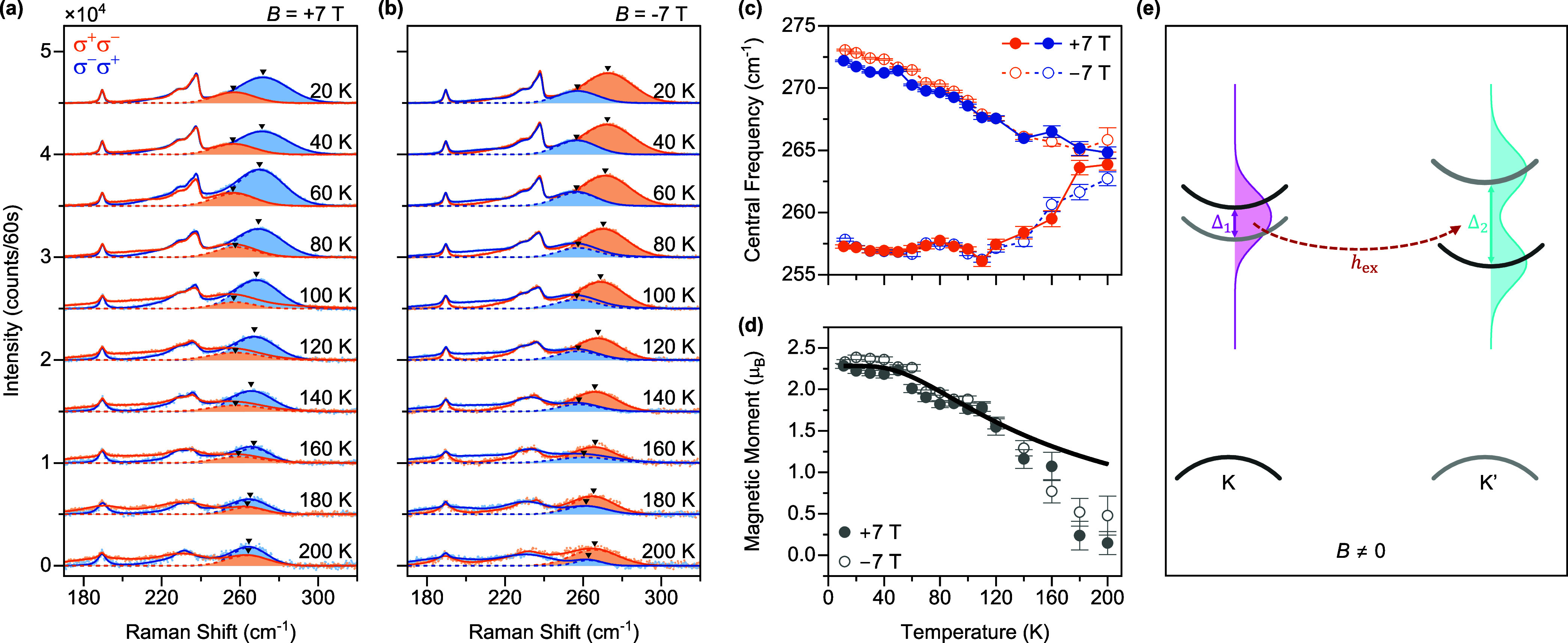
Temperature dependence of effective phonon magnetic moment. Raman spectra in the helicity-switched (σ^+^σ^–^/σ^–^σ^+^) channels as a function of temperature acquired in (a) +7 T and (b) −7 T magnetic field. The spectra are vertically offset for clarity. (c) Frequencies of the split Ω_ph_^±^ in +7 T (solid circles) and −7 T (empty circles) magnetic field as a function of temperature. (d) Evolution of phonon magnetic moment in the units of μ_B_ as a function of temperature. The tanh function predicted by the orbital-phonon coupling model is overlaid for comparison. (e) Schematic of the impact of thermal broadening on the nondegenerate orbital transitions. As the temperature increases, the thermal broadening of the electronic bands overwhelms the low-energy orbital transition (Δ_1_), while the high-energy orbital transition (Δ_2_) is still well-defined. As a result, the Δ_1_ orbital transition is annihilated and recreated in the opposite valley through the exchange interaction (*h*_ex_).

Up to here, we have established that the picture of orbital-phonon coupling can reproduce the effective magnetic moment associated with the circularly polarized *E*″ phonon in MoS_2_. Note that this model was used to explain phonon magnetic moments in 4*f* paramagnet CeCl_3_ and 3*d* magnet CoTiO_3_.^26^ For both cases, the splitting of hybridized orbital-phonon states relies on the population imbalance between the split energy levels, which produces net spin polarization. Specifically, in the paramagnetic phase, the net spin polarization depends on the magnetic field and temperature through tanh(*g*μ_B_*B/k*_B_*T*), where *g* is the g-factor of the Kramers states.^26^ To apply the orbital-phonon coupling model to MoS_2_, which is a nonmagnetic material, an important question to be addressed is the origin of the net spin polarization. Therefore, we carry out temperature-dependent measurements at a finite magnetic field to extract the dependence of the effective phonon magnetic moment on *B* and *T*. Figure 2a shows the Raman spectra in the σ^+^σ^–^ and σ^–^σ^+^ channels acquired at a +7 T magnetic field as a function of temperature. As the material warms from 12 K, the split circularly polarized phonon modes gradually merge and finally overlap at 200 K. Under the −7 T magnetic field, the spectra in the σ^+^σ^–^ and σ^–^σ^+^ channels exchange (see Figure 2b) as they are connected by a time-reversal operation.

Strikingly, the temperature dependence of the split Ω_ph_^+^ and Ω_ph_^–^ modes are different. As shown in Figure 2c, at ±7 T, the higher-frequency Ω_ph_^+^ mode as a function of temperature largely follows the anharmonic decay like a typical phonon, while the lower-frequency Ω_ph_^–^ mode rapidly merges toward the higher-frequency one when temperature is above 110 K. Based on the orbital-phonon coupling model under finite external magnetic field (see Figure 1f), the Ω_ph_^+^ and Ω_ph_^–^ modes are derived from the Δ_2_ and Δ_1_ orbital transitions (Δ_2_ > Δ_1_), respectively. The thermal broadening of the split conduction bands will primarily impact the lower-energy orbital transition (Δ_1_), while the higher-energy orbital transition (Δ_2_) is more robust. As sketched in Figure 2e, when the elevated temperature annihilates the low-energy orbital transition in one valley, it can be recreated in the opposite valley through the exchange interaction (*h*_ex_). As *h*_ex_ = 9 meV^40^ is approximately 105 K, it is consistent with the temperature (110 K) when the Ω_ph_^–^ mode starts merging toward the Ω_ph_^+^ mode. Figure 2d shows the effective phonon magnetic moments as a function of temperature, which saturates at ∼2.4 μ_B_ at 12 K. The temperature dependence of effective phonon magnetic moments predicted by the orbital-phonon coupling model follows a tanh function, which captures the trend in the 12–120 K range. Above 120 K, the experimental data deviates from the tanh function due to the vanishing of the resonance with the *A* exciton as the temperature approaches 200 K.^28^

**Figure 3:**
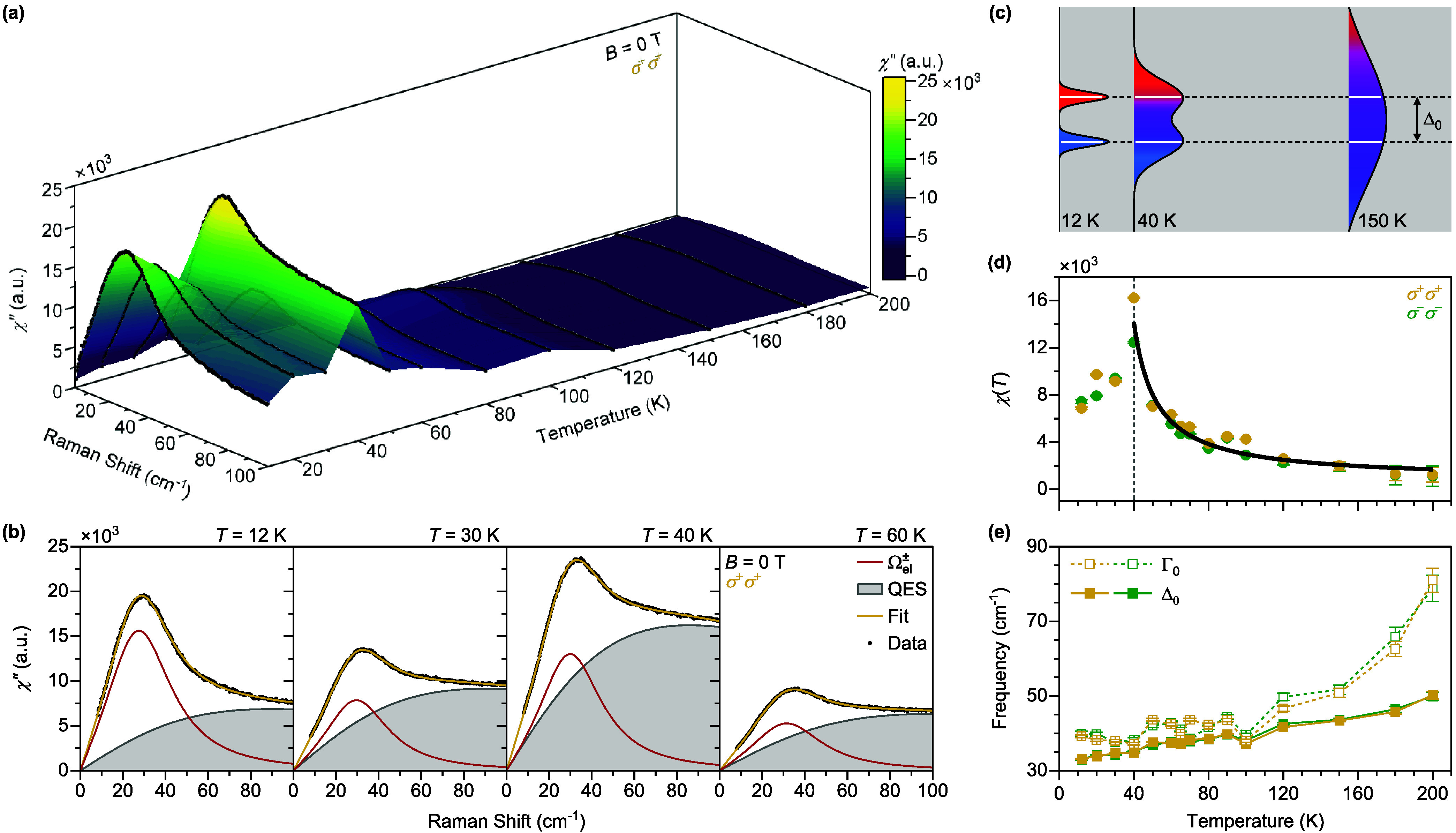
Contribution of spin fluctuations to the Raman response. (a) Raman response χ″ in the helicity-conserved σ^+^σ^+^ channel as a function of temperatures. (b) Fit to the Raman response at selected temperatures using eq 2. The red curve is the orbital transition peak and the gray broad continuum is the QES. (c) Schematic of the evolution of the mixture of the conduction bands with opposite spins at different temperatures. (d) Temperature evolution of the QES spectral weight χ(*T*) extracted using eq 3. The black solid curve is the fit to the Curie–Weiss law. (e) Central frequency (solid squares) and line width (empty squares) of the orbital transition peak as a function of temperature.

Based on the thermodynamic behavior discussed above, we propose that the resonant excitation of the *A* exciton pumps the electrons to the unoccupied conduction bands, enabling the subsequent orbital transitions between the spin–orbit split conduction bands. Such orbital transition may be subject to sizable spin fluctuations when the thermal broadening leads to a mixture of conduction bands with opposite spins. Figure 3a shows the Raman response χ″(ω) at different temperatures in the absence of a magnetic field. The broad continuum extending to 100 cm^–1^ in the helicity-conserved σ^+^σ^+^ channel can be ascribed to quasi-elastic scattering (QES), which is typically from magnetic fluctuations in low-dimensional spin systems.^41−44^ The QES signal is especially noticeable at around 40 K. Similar results are also obtained in the σ^–^σ^–^ channel (see Supporting Information Section 2). Figure 3b shows the fittings to the Raman response at selected temperatures, in which the orbital transition peak is modeled by a damped Lorentzian profile and the QES is expressed as a Lorentzian profile
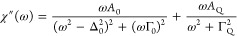
2where *A*_0_, Δ_0_, and Γ_0_ are the amplitude, frequency, and damping rate of the orbital transition, respectively, while *A*_Q_ and Γ_Q_ represent the amplitude and damping rate of QES, respectively. We extract the QES spectral weight (or integrated intensity I.I.) using the Kramers–Kronig relation
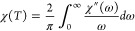
3where we choose the high-energy cutoff of the integral to be 100 cm^–1^. As shown in Figure 3d, χ(*T*) exhibits a diverging behavior from 200 to 40 K, which can be fitted to a Curie–Weiss law.^45,46^ Therefore, we attribute the QES to the spin fluctuations, and χ(*T*) resembles the magnetic susceptibility behavior of a paramagnet. As sketched in Figure 3c, at the lowest temperatures where *k*_*B*_*T* ≪ Δ_0_, the conduction bands with opposite spins are well separated, while at high temperatures where *k*_*B*_*T* ≫ Δ_0_, the conduction bands are fully overwhelmed by the thermal broadening. At 40 K where *k*_B_*T* ≈ Δ_0_, the susceptibility diverges, indicating that the spins in the conduction bands have the maximum fluctuations. We speculate that the evolution of susceptibility may point to a phase transition at 40 K. While detailed theoretical calculations are needed to investigate this possibility, we can use the Curie–Weiss law to account for the paramagnetic phase above 40 K. Concurrent with the diverging QES intensity, the orbital transition peak is also significantly enhanced. As shown in Figure 3e, the central frequency of the orbital transition gradually decays with decreasing temperature, confirming that this is not a phonon mode. Also, the line width of the orbital transition is comparable with the central frequency below 100 K and then rapidly diverges, resembling the behavior of a heavily damped electronic state.

**Figure 4:**
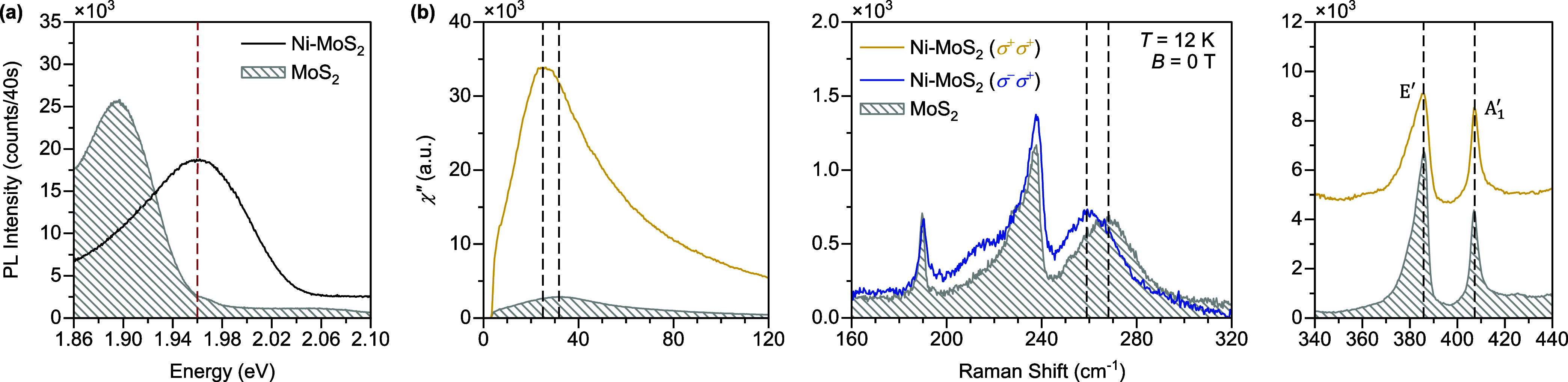
Tunability of orbital-phonon hybridized states. (a) Photoluminescence (PL) of Ni-MoS_2_ (black curve) and MoS_2_ (gray shadow) acquired using 532 nm excitation laser under the same measurement condition. The vertical red dashed line marks the location of 1.96 eV laser line (633 nm) used in our Raman measurements. (b) Comparison of Raman response between Ni-MoS_2_ and MoS_2_ in the frequency ranges containing orbital transition mode (0–120 cm^–1^), *E*″ phonon mode (160–320 cm^–1^), and *E*′ and *A*_1_^′^ phonon modes (340–440 cm^–1^), respectively. For a fair comparison between Ni-MoS_2_ and MoS_2_, the Raman spectra in the σ^+^σ^+^ channel are normalized to the *A*_1_^′^ phonon mode, while the spectra in the σ^–^σ^+^ channel are normalized to the 190 cm^–1^ mode. The Raman spectra in the 340–440 cm^–1^ range are vertically offset for clarity. All of the Raman and PL data were taken at *T* = 12 K and *B* = 0 T.

Finally, we tune the orbital-phonon hybridized states through the optimization of the laser resonance. Deposition of nanometer-thickness metal overlayers has been demonstrated as an effective method of modifying electronic and structural properties of MoS_2_.^47−49^ Here, we deposited 4 nm of nickel thin films on monolayer MoS_2_ (denoted as Ni–MoS_2_), and we find that the *A* exciton shifts to 1.96 eV (see photoluminescence data in Figure 4a), which perfectly overlaps with the 633 nm laser for Raman measurements. As a result, the orbital transition mode is enhanced by a factor of 12 compared with MoS_2_ (see the left panel of Figure 4b). Also, the frequency of the orbital transition mode shifts toward low frequency by ∼6 cm^–1^. Concurrently, the circularly polarized phonon mode red-shifts by nearly the same amount (see the middle panel of Figure 4b), confirming the orbital transition and circularly polarized phonon are indeed coupled. Note that all of the other phonon modes remain unchanged (see the right panel of Figure 4b), which suggests that Ni layers only modify the electronic structure of MoS_2_ while having little impact on the lattice. Moreover, the circularly polarized phonon mode in Ni-MoS_2_ has comparable intensity with that of MoS_2_, possibly because the phonon energy is much larger than that of the orbital transition.

It is worth noting that the unpaired spins associated with defects in two-dimensional materials may lead to magnetic phenomena.^50^ However, this type of disorder-induced magnetism can be ruled out in our case for the following reasons. First, as shown in our previous work,^28^ the giant magnetic moments of the circularly polarized phonon are also observed in high-quality exfoliated MoS_2_ samples with very low defect density. Second, the magnetic responses we observed, including phonon Zeeman splitting and diverging QES at 40 K appear only when the laser photon energy is in resonance with the intrinsic conduction bands of MoS_2_. No defect states are involved in this process.

## Conclusions

In conclusion, we investigated the helicity-resolved magneto-Raman response emerging from the lattice, orbital, and spin degrees of freedom in monolayer MoS_2_. The resonant excitation of the *A* exciton enables an orbital transition between the spin–orbit split conduction bands, which have sizable coupling with the circularly polarized phonon mode. The split conduction bands have strong spin fluctuations that lead to a population imbalance between the split energy levels, mimicking paramagnetic behavior. The resultant orbital-phonon hybridized modes exhibit a large effective phonon magnetic moment that can be precisely reproduced by our model. Our work establishes a generalized picture of creating large effective phonon magnetic moments in a spin-valley coupled nonmagnetic semiconductor, which may potentially be used in photon-induced sensor applications.

## Methods

### Samples

Monolayer MoS_2_ was synthesized using chemical vapor deposition on Si/SiO_2_ substrate as described in ref (51). Deposition of nickel thin films on MoS_2_ occurred at room temperature using a 2 mm nickel wire (99.995% pure) in a mini electron-beam evaporator (MANTIS QUAD-EV) chamber with a base pressure of 4 × 10^–10^ Torr.^52^ A flux monitor was used to maintain a consistent deposition rate of 2.5 Å/min. The thickness of nickel on MoS_2_ is about 4 nm to ensure full coverage.

### Raman Spectroscopy

Raman spectra were carried out using a Horiba LabRAM HR Evolution Raman microscope in a backscattering configuration. A schematic of the helicity-resolved Raman setup is shown in the Supporting Information Section 3. A 633 nm CW laser was used for taking Raman spectra because its photon energy is close to that of the *A* exciton in monolayer MoS_2_. The laser light was focused on a spot with a 2–3 μm diameter using a 40× objective lens. The scattered light was dispersed by an 1800 grooves/mm grating and detected by a thermoelectrically cooled CCD. The laser power was kept below 0.5 mW. For photoluminescence measurements, a 532 nm laser with a power of 0.5 mW and a 600 grooves/mm grating were used. Samples were mounted in a closed-cycle helium cryostat with windows for optical access. The variable-temperature cryostat was interfaced with a cryogen-free magnetic field from Cryo Industries of America, which provides an out-of-plane magnetic field up to 7 T. All of the Raman and photoluminescence measurements were conducted in a vacuum with base pressure below 7 × 10^–7^ Torr.

## Data Availability

The data underlying this study are openly available in Zenodo at https://doi.org/10.5281/zenodo.14531470.

## Supporting Information Available

First-principles calculation of electron–phonon coupling strength; low-frequency Raman data in the σ^–^σ^–^ channel; and schematic of the helicity-resolved Raman spectroscopy setup (PDF)
