# Supplementary material for: Origin of Large Effective Phonon Magnetic Moments in Monolayer MoS2
Source: ACS Nano. 2025 Mar 13;19(11):11241–8. doi: 10.1021/acsnano.4c18906 (PMC11948479; doi:10.1021/acsnano.4c18906)
Supplement: Supplementary file 1 — nn4c18906_si_001.pdf [file nn4c18906_si_001.pdf]

Supporting Information

Origin of Large Effective Phonon Magnetic Moments in Monolayer MoS_2

Hussam Mustafa,[1,*] Cynthia Nnokwe,[2,*] Gaihua Ye,[2] Mengqi Fang,[3]
Swati Chaudhary,[4] Jia-An Yan,[5] Kai Wu,[2] Connor J. Cunningham,[6]
Colin M. Hemesath,[6] Andrew James Stollenwerk,[6] Paul M. Shand,[6]
Eui-Hyeok Yang,[3] Gregory A. Fiete,[7,8,9] Rui He,[2,†] and Wencan Jin[1,‡]

[1]*Department of Physics, Auburn University, Auburn, Alabama 36849, USA*

[2]*Department of Electrical and Computer Engineering,*
Texas Tech University, Lubbock, Texas 79409, USA

[3]*Department of Mechanical Engineering,*
Stevens Institute of Technology, Hoboken, New Jersey 07030, USA

[4]*Institute for Solid State Physics, The University of Tokyo, Chiba 277-8581, Japan*

[5]*Department of Physics, Astronomy, and Geosciences,*
Towson University, Towson, Maryland 21252, USA

[6]*Department of Physics, University of Northern Iowa, Cedar Falls, Iowa 50614, USA*

[7]*Department of Physics, Northeastern University,*
Boston, Massachusetts 02115, USA

[8]*Quantum Sensing and Materials Institute,*
Northeastern University, Burlington, Massachusetts 01803, USA

[9]*Department of Physics, Massachusetts Institute of Technology,*
Cambridge, Massachusetts 02139, USA

(Dated: March 7, 2025)

Contents

S1. First-principles calculation of electron-phonon coupling strength 3

S2. Low-frequency Raman data in the $\sigma^-\sigma^-$ channel. 4

S3. Helicity-resolved Raman spectroscopy setup 5

References 6

S1. First-principles calculation of electron-phonon coupling strength

First-principles calculations were carried out to generate the electronic and phonon band structures of monolayer MoS_2 with spin-orbit coupling (SOC) using Quantum ESPRESSO [1, 2] in PBE functional [3]. Norm-conserving pseudopotentials for Mo and S were adopted [4, 5]. The planewave cutoff was set to be 70 Ryd and k-point mesh grid was set to be $12 \times 12 \times 1$.

We then employed the EPW code [6, 7] to calculate the strength of the electron-phonon coupling matrix elements. Specifically, the coupling strength between the phonon and the orbital transition can be evaluated using the Hamiltonian:

$$H_{\text{el-ph}} = (a^\dagger + a)\hat{O}_a + (b^\dagger + b)\hat{O}_b$$

where the operators $\hat{O}_{a/b}$ can be written as

$$\hat{O}_a = g_a|\psi_1\rangle\langle\psi_3| - g_a^*|\psi_2\rangle\langle\psi_4| + \text{h.c.}$$
$$\hat{O}_b = g_b|\psi_1\rangle\langle\psi_3| - g_b^*|\psi_2\rangle\langle\psi_4| + \text{h.c.}$$

The electron-phonon coupling strength ($\tilde{g}$) between the Γ-point phonon modes and the orbital transition (Δ_0) are listed in the Table.

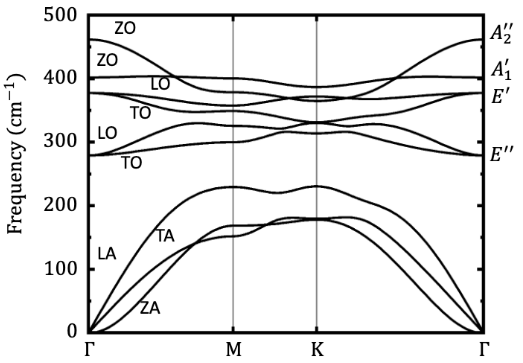

Phonon mode \ Orbital transition	Δ_0 (4 meV)
A_2'' (56.9 meV)	2.8×10^{-7} meV
A_1' (49.2 meV)	1.2×10^{-6} meV
E' (46.3 meV)	8.2×10^{-7} meV
E'' (34.2 meV)	8.0 meV

FIG. S1: Calculated phonon band dispersion and the electron-phonon coupling strength between the Δ_0 orbital transition and the A_2'', A_1', E', and E'' phonon modes.

S2. Low-frequency Raman data in the $\sigma^-\sigma^-$ channel.

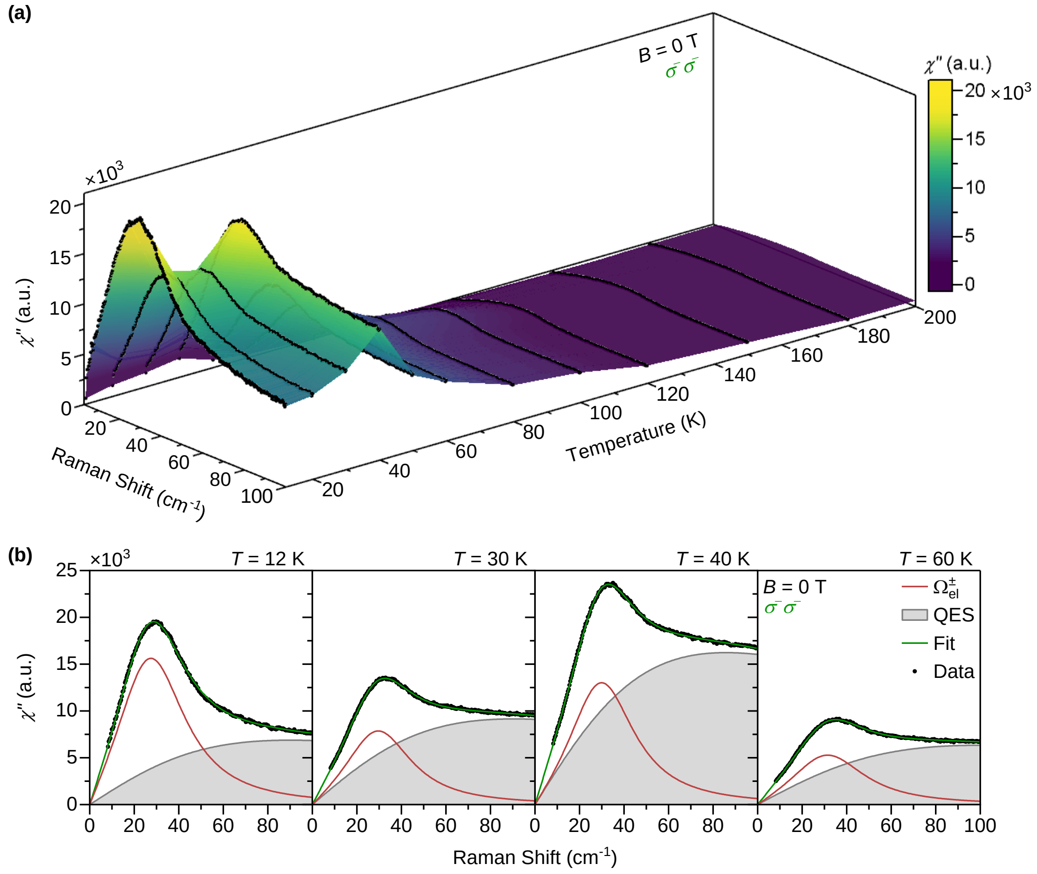

FIG. S2: **(a)** Raman response χ'' in the helicity-conserved $\sigma^-\sigma^-$ channel as a function of temperature. Similar plot of the $\sigma^+\sigma^+$ channel is shown in the main text Figs. 3(a) and (b). **(b)** Fit to the Raman response at selected temperatures using main text Eq.(2). The red curve is the orbital transition peak and the gray broad continuum is the QES.

S3. Helicity-resolved Raman spectroscopy setup

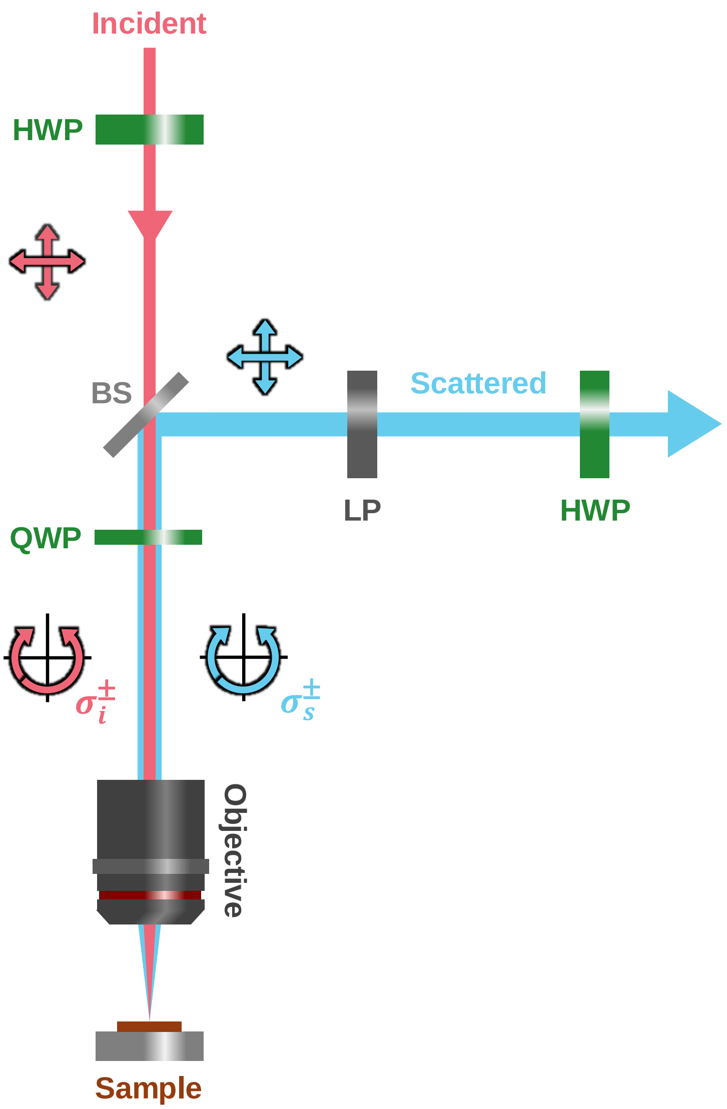

FIG. S3: Schematic diagram of the experimental setup of helicity-resolved Raman spectroscopy. The incident laser (pink) passes through a half waveplate (HWP) and a quarter waveplate (QWP) to produce a left- (σ^+) or right-handed (σ^-) circularly polarized light. The Raman scattered light (blue) passes through the same QWP and propagates to the detection path. A linear polarizer (LP) selects the helicity of the scattered light. To achieve consistent detection efficiency, the selected polarization is rotated by an HWP to align with the grating grooves of the spectrometer. BS: Beam splitter.

* These two authors contributed equally

† rui.he@ttu.edu

‡ wjin@auburn.edu

[1] P. Giannozzi, S. Baroni, N. Bonini, M. Calandra, R. Car, C. Cavazzoni, D. Ceresoli, G. L. Chiarotti, M. Cococcioni, I. Dabo, *et al.*, Journal of Physics: Condensed Matter **21**, 395502 (2009).

[2] P. Giannozzi, O. Andreussi, T. Brumme, O. Bunau, M. B. Nardelli, M. Calandra, R. Car, C. Cavazzoni, D. Ceresoli, M. Cococcioni, *et al.*, Journal of Physics: Condensed Matter **29**, 465901 (2017).

[3] J. P. Perdew, K. Burke, and M. Ernzerhof, Physical Review Letters **77**, 3865 (1996).

[4] D. Hamann, Physical Review B **88**, 085117 (2013).

[5] M. Schlipf and F. Gygi, Computer Physics Communications **196**, 36 (2015).

[6] F. Giustino, M. L. Cohen, and S. G. Louie, Physical Review B **76**, 165108 (2007).

[7] H. Lee, S. Poncé, K. Bushick, S. Hajinazar, J. Lafuente-Bartolome, J. Leveillee, C. Lian, J.-M. Lihm, F. Macheda, H. Mori, *et al.*, npj Computational Materials **9**, 156 (2023).
